# Prevalence and phenotypic impact of rare potentially damaging variants in autism spectrum disorder

**DOI:** 10.1186/s13229-021-00465-3

**Published:** 2021-10-06

**Authors:** Behrang Mahjani, Silvia De Rubeis, Christina Gustavsson Mahjani, Maureen Mulhern, Xinyi Xu, Lambertus Klei, F. Kyle Satterstrom, Jack Fu, Michael E. Talkowski, Abraham Reichenberg, Sven Sandin, Christina M. Hultman, Dorothy E. Grice, Kathryn Roeder, Bernie Devlin, Joseph D. Buxbaum

**Affiliations:** 1 Seaver Autism Center for Research and Treatment, Icahn School of Medicine at Mount Sinai, New York, NY 10029 USA; 2 Department of Psychiatry, Icahn School of Medicine at Mount Sinai, New York, NY USA; 3 Department of Medical Epidemiology and Biostatistics, Karolinska Institutet, Stockholm, Sweden; 4 Friedman Brain Institute, Icahn School of Medicine at Mount Sinai, New York, NY USA; 5 The Mindich Child Health and Development Institute, Icahn School of Medicine at Mount Sinai, New York, USA; 6 Department of Psychiatry, University of Pittsburgh School of Medicine, Pittsburgh, PA USA; 7 Stanley Center for Psychiatric Research, Broad Institute of MIT and Harvard, Cambridge, MA USA; 8 Program in Medical and Population Genetics, Broad Institute of MIT and Harvard, Cambridge, MA USA; 9 Center for Genomic Medicine, Massachusetts General Hospital, Boston, MA USA; 10 Department of Neurology, Massachusetts General Hospital and Harvard Medical School, Boston, MA USA; 11 Department of Statistics, Carnegie Mellon University, Pittsburgh, PA USA; 12 Department of Genetics and Genomic Sciences, Icahn School of Medicine at Mount Sinai, New York, NY USA; 13 Department of Neuroscience, Icahn School of Medicine at Mount Sinai, New York, NY USA

**Keywords:** Autism spectrum disorder, Copy number variant, Single nucleotide variant, Intellectual disability, Whole exome sequencing, PAGES

## Abstract

**Background:**

The Autism Sequencing Consortium identified 102 high-confidence autism spectrum disorder (ASD) genes, showing that individuals with ASD and with potentially damaging single nucleotide variation (pdSNV) in these genes had lower cognitive levels and delayed age at walking, when compared to ASD participants without pdSNV. Here, we made use of a Swedish sample of individuals with ASD (called PAGES, for Population-Based Autism Genetics & Environment Study) to evaluate the frequency of pdSNV and their impact on medical and psychiatric phenotypes, using an epidemiological frame and universal health reporting. We then combine findings with those for potentially damaging copy number variation (pdCNV).

**Methods:**

SNV and CNV calls were generated from whole-exome sequencing and chromosome microarray data, respectively. Birth and medical register data were used to collect phenotypes.

**Results:**

Of 808 individuals assessed by sequencing, 69 (9%) had pdSNV in the 102 ASC genes, and 144 (18%) had pdSNV in the 102 ASC genes or in a larger set of curated neurodevelopmental genes (from the Deciphering Developmental Disorders study, the gene2phenotype database, and the Radboud University gene lists). Three or more individuals had pdSNV in *GRIN2B*, *POGZ*, *SATB1*, *DYNC1H1*, *SCN8A,* or *CREBBP*. In comparison, out of the 996 individuals from whom CNV were called, 105 (11%) carried one or more pdCNV, including four or more individuals with CNV in the recurrent 15q11q13, 22q11.2, and 16p11.2 loci. Carriers of pdSNV were more likely to have intellectual disability (ID) and epilepsy, while carriers of pdCNV showed increased rates of congenital anomalies and scholastic skill disorders. Carriers of either pdSNV or pdCNV were more likely to have ID, scholastic skill disorders, and epilepsy.

**Limitations:**

The cohort only included individuals with autistic disorder, the more severe form of ASD, and phenotypes are defined from medical registers. Not all genes studied are definitively ASD genes, and we did not have de novo information to aid in classification.

**Conclusions:**

In this epidemiological sample, rare pdSNV were more common than pdCNV and the combined yield of potentially damaging variation was substantial at 27%. The results provide compelling rationale for the use of high-throughout sequencing as part of routine clinical workup for ASD and support the development of precision medicine in ASD.

**Supplementary Information:**

The online version contains supplementary material available at 10.1186/s13229-021-00465-3.

## Background

Autism spectrum disorder (ASD) is a childhood-onset neurological and developmental disorder that affects more than 1% of the population [1]. The affected individuals can have lifelong impairments in social interaction, communication, and adaptive functioning. In the Diagnostic and Statistical Manual of Mental Disorders, 4th Edition (DSM-IV) [2], severity across the ASD spectrum was reflected by different terms, from a mild form called Asperger's syndrome, to the severest form called autistic disorder. In 2013, in the Diagnostic and Statistical Manual of Mental Disorders, Fifth Edition (DSM-5) [3], autistic disorder, Asperger's syndrome and additional pervasive developmental disorder diagnoses were replaced with the umbrella diagnosis of ASD, with severity specifiers for social communication and for restricted interests and repetitive behaviors.

ASD has a complex genetic architecture with both rare and common variation contributing to risk. While common variation accounts for the majority of genetic liability for autism, rare variation, often de novo*,* accounts for substantial individual liability [4]. Numerous studies have identified de novo and inherited SNV and CNV associated with ASD [5–7]. Given that CNV has been easier to identify in both affected and unaffected populations, there is a large literature describing medical findings associated with CNV, as well as reliable estimates of the frequency of potentially damaging copy number variation (pdCNV) [5, 8–17]. Less is known about potentially damaging single nucleotide variation (pdSNV).

In the most extensive whole-exome sequencing study to date, the Autism Sequencing Consortium (ASC) identified 102 genes that, when carrying specific types of deleterious variants, are strongly associated with risk for ASD [13]. The same study showed that individuals with ASD and pdSNV in these 102 genes showed lower IQs and greater delays in walking, on average, as compared to individuals with ASD without pdSNV. However, clinical information was restricted in that study. Over 5% of all ASD participants carried pdSNV, although the ascertainment of the cohorts that make up the ASC study were almost universally convenience samples, so these rates are hard to generalize.

The objective of the current work is to extend the comparison of comorbid medical findings in individuals diagnosed with ASD with or without pdSNV or pdCNV, making use of a Swedish epidemiological sample called Population-Based Autism Genetics and Environment Study (PAGES) [18]. By incorporating robust and relatively unbiased phenotype data obtained from the Swedish national register, we compare the phenotypes of those with ASD with potentially damaging variation (PDV)—pdCNV or pdSNV, and those with ASD without a PDV. In addition, because sample collection was carried out in an epidemiological framework, we are able to describe the genetic architecture of PDV in ASD on a population level, including estimates of rates of genetic findings in ASD. In the companion study by Klei et al. [19], the inter-related role of common variation and PDV in ASD risk is explored in the PAGES sample.

## Methods

### Study population

In this study, we used data collected from study participants in PAGES, a large ongoing population-based cohort study in Sweden that started in 2012 with the overall aim to identify possible genetic and environmental risk factors for ASD [4]. The study was approved by the Regional Ethical Review Board in Stockholm, Sweden, and the Institutional Review Board at the Icahn School of Medicine at Mount Sinai, New York, USA. All individuals with a diagnosis of ASD according to the International Classification of Diseases (ICD) 9 and 10 criteria were identified in the Swedish National Patient Register. Our focus here is on autistic disorder, defined by ICD-9 codes 299.A/B/X and ICD-10 code F84.0. The eligible individuals were born in Sweden between 1960 and 1996 and followed up through 2011.

In PAGES, after a potential case was identified in the Swedish National Patient Register and the diagnosis confirmed, research nurses informed the family about the genetic study with a letter followed up with a phone call. Those interested in participating provided informed consent and biospecimens (blood in most cases). Information about sex, age at the time of diagnosis, date of admission and discharge, and diagnostic codes for intellectual functioning and psychiatric comorbidities were extracted from the Swedish National Patient Register after the consent form was signed. The date of the first registered ASD diagnosis was used as the diagnosis date.

In addition to the Swedish National Patient Register, the Multi-generation Register was also accessed, which allowed for the identification of family relations, as was the Swedish Medical Birth Register, which contained birth characteristics of all Swedish-born children since 1973 (including prenatal, perinatal and neonatal variables). For more information about the Swedish national registers, see [20].

DNA from 827 PAGES participants with ASD was subjected to whole exome sequencing by ASC [13]. In addition, 1,154 PAGES ASD samples were genotyped on either Infinium OmniExpress Exome V1 (*n* = 239, number of single nucleotide polymorphisms (SNPs): 951,117), V1.1 (*n* = 152, number of SNPs: 958,178), V1.2 (*n* = 553, number of SNPs: 964,193), V1.4 (*n* = 219, number of SNPs: 960,919), or the Infinium Global Screening Array (*n* = 82, number of SNPs: 700,078).

### SNV calling

SNV was called using the Genome Analysis Toolkit [21] HaplotypeCaller package version 3.4 (for more details, see [13]). Rare SNV was defined as those absent from Genome Aggregation Database (gnomAD). Rare SNV in likely ASD and intellectual disability (ID) genes was classified as potentially damaging if the variant was either (1) a protein-truncating variant, or (2) a missense variant with a "Missense badness, PolyPhen-2, Constraint" (MPC) score > 2 [22].

While we initiated this study to extend the genome-wide ASC results, we recognize that the ASC gene list is both incomplete and will also include small numbers of false-positive findings. For this reason, and in response to reviews, we created a larger set of curated genes involved in ASD and/or other neurodevelopmental disorders. We made use of multiple data sources to define potential ASD genes. First, we used the 102 genes reported by the ASC in Satterstrom et al. [13]. Second, we created a developmental delay/ID gene list, relying on three sources of data. We began by incorporating the 94 genes reported in the Deciphering Developmental Disorders study in 2017 [23]. In addition, we accessed the gene2phenotype developmental disorders (DD) gene list [24] from [25] and the Radboud University Medical Center ID gene panel (version DG 2.18) from [26] on January 26, 2021. For these latter two gene lists, biallelic and imprinted genes were removed, and only genes with autosomal dominant or X-linked inheritance that were found in both lists were included in a combined list, to focus on genes clearly involved in neurodevelopmental disorders. The combined list from the Deciphering Developmental Disorders study, gene2phenotype, and Radboud University Medical Center is referred to as DGR, and included 560 genes (Additional File 1: Table S1). Hence, results are presented for three gene lists: (1) the 102 genes identified by the ASC (ASC102), (2) the independently derived but overlapping DGR gene list (n = 560); and (3) the union of the above two lists (ASC102 + DGR; n = 597 genes). We summarize findings for pdSNV in these gene lists.

### CNV calling

CNV calls were generated from 1154 ASD samples genotyped on the Infinium OmniExpress Exome by PennCNV using hg19 genomic coordinates. Data and calls were cleaned using standard procedures in PennCNV (B Allele Frequency drift ≤ 0.01, |waviness factor |≤ 0.05, log R ratio SD ≤ 0.3). We combined neighboring CNV if the gap between them was less than or equal to 20% of the total length of the two adjacent CNV plus the gap. We excluded CNV with SNPs < 20, as well as CNV with at least 50% reciprocal overlap with previously described common CNV regions according to the Database of Genomic Variants v10.

We first developed a list of CNV that had prior strong evidence for being associated with a genomic neurodevelopmental disorder. To generate this list, we used curated lists of CNV from ClinGen and DECIPHER. We accessed the ClinGen ftp site [27] and downloaded the region curation list for hg19. We merged this list with the list from ClinGen Dosage Sensitivity Curation Page [28]. We chose the regions with haploinsufficiency and/or triplosensitivity scores of 3 (sufficient evidence) and treated deletions and duplications separately wherever indicated. For DECIPHER, we downloaded the list of CNV syndromes from [29]. We excluded those with a grade of 3 (susceptibility locus) and treated deletions and duplications separately wherever indicated. We then merged these lists (see Additional file 3: Table S2 for the final list). For a few regions with discrepant classifications in the two databases, we used the ClinGen classification.

We called CNV potentially damaging if it satisfied one or more of the following three conditions: (1) if the CNV occurred within a locus associated with known genomic disorders curated by ClinGen and/or DECIPHER (as noted above); (2) if the CNV was larger than 3 Mb; or, (3) if the CNV was larger than 1 Mb and included one or more coding exons from at least one brain-expressed gene (as determined from the UCSC Genome Browser). For chromosome X, we included only known loci associated with genomic disorders due to potentially lower quality of CNV calls from the sex chromosomes [30]. Five individuals with evidence for three or more large CNV (> 1 Mb) were removed due to concerns about the quality of the sample. In addition, we removed any sample with a called CNV > 45 Mb, eliminating one individual with a CNV of 75 Mb. The 45 Mb threshold was derived from an ongoing analysis by the GATK Team at Broad Institute to generate CNV calls for ASD samples (including PAGES) from WES data using gCNV [31]. In the GATK calls, pdCNV status for 77% of the variants associated with known genomic disorders from this study was confirmed, and no CNV in autosomes was larger than 45 Mb. We retained 996 high-quality samples for further analyses.

Note that the American College of Medical Genetics and Genomics (ACMG) guidelines were not used in this study to classify damaging variants (CNV or SNV) [32, 33]. Some of the variants that we classified as potentially damaging could be variants of uncertain significance based on ACMG guidelines. In addition, we did not have de novo information to aid in classification.

### Phenotypic information

We extracted information for the following variables from the Swedish National Patient Register and the Swedish National Birth Register: ID (IQ < 70), attention-deficit/hyperactivity disorder (ADHD), psychotic disorders (schizophrenia, schizotypal, delusional, and other non-mood psychotic disorders), obsessive–compulsive disorder (OCD), anxiety disorder, speech and language disorders, scholastic skill disorders, motor function disorders, epilepsy, sleeping disorders, hypotonia, birth defects, prenatal growth rate, gestational age in weeks, weight, height and head circumference at birth, and Apgar scores (Additional file 2: Table S3). Thirteen individuals with a diagnosis of Down syndrome and one with a diagnosis of Turner’s syndrome were not included in downstream analyses. (More broadly, individuals with sex chromosome aneuploidies were excluded from PAGES at the time of recruitment.)

The average head circumference of healthy newborns is 33–35 cm [34]. While the range depends on the length of the newborn, among other attributes, we used head circumference without adjustment, defining "HC-small" if the circumference was smaller than 32 cm and "HC-large" if it was larger than 38. Small for gestational age was defined as birth weight less than two standard deviations below the mean using Swedish growth charts [35], while large for gestational age was defined as birth weight more than two standard deviations above the mean.

### Statistical analysis

To identify comorbidities and birth characteristics associated with ASD probands who carry damaging mutations, we used a logit model in which carrier status of the damaging variant type was the dependent variable (carrier of pdCNV or pdSNV or not) and predictors were sex, used as a covariate, and potential comorbidity or characteristic. Thus, a series of models were fit, one for each potentially associated feature. We reported the resulting odds ratio (OR), *p* values, and 95% confidence intervals (CIs) for the OR after adjusting for the sex variable.

## Results

### Demographic data

After quality control, whole-exome sequencing (WES) data were available for 808 probands, and genotype (chromosomal microarray or CMA) data were available for 996 probands (Table 1). Of these individuals, 70% were male (Table 1).

**Table 1:** Genetic characterization of probands

	CMA probands	WES probands	CMA and WES probands
Total (*n*)	996	808	674
Average diagnosis age (SD)	8.2 (8.4)	7.9 (8.3)	8.0 (8.5)
Females (*n*, (%))	298 (30%)	229 (28%)	185 (27%)
Average diagnosis age (SD)	8.8 (9.2)	8.6 (8.9)	9.0 (9.5)
Males (*n*, (%))	698 (70%)	579 (72%)	489 (72%)
Average diagnosis age (SD)	7.9 (8.1)	7.6 (8.0)	7.6 (8.1)

Of the comorbidities and birth characteristics for the population of ASD probands (Table 2), ID was most common (48%), and epilepsy was second (31%). Individuals with congenital anomalies had the lowest age of ASD diagnosis, while individuals with psychotic disorders had the highest age of ASD diagnosis. Comorbidities and birth characteristics of the probands which were not genotyped or sequenced are presented in Table S4 (Additional file 2).

**Table 2:** Demographics of comorbidities and birth characteristics of probands

Birth characteristics and comorbidities	CMA probands (*n* = 996)		WES probands (*n* = 808)	
	*n* (%)	Average diagnosis age (y) for ASD (SD)	*n* (%)	Average diagnosis age (y) for ASD (SD)
HC—large^1^	40 (5%)	7.9 (7.3)	30 (5%)	8.4 (7.7)
HC—small^2^	87 (11%)	4.6 (5.9)	73 (11%)	4.0 (4.4)
Large for gestational age^3^	38 (5%)	7.6 (7.7)	30 (4%)	5.2 (5.7)
Small for gestational age^4^	37 (5%)	4.1 (6.1)	35 (5%)	4.3 (6.3)
Congenital anomalies	74 (11%)	2.9 (4.9)	57 (10%)	3.1 (4.7)
Motor function disorders	55 (5%)	4.8 (5.0)	43 (5%)	4.6 (4.8)
Scholastic skill disorders	155 (16%)	5.1 (5.6)	121 (15%)	5.2 (6.2)
Speech/language disorders	133 (13%)	4.8 (5.8)	102 (13%)	4.6 (5.1)
ADHD	203 (20%)	8.4 (7.4)	160 (20%)	7.7 (7.2)
Anxiety disorder	29 (3%)	8.8 (6.5)	20 (2%)	9.5 (7.0)
Epilepsy	309 (31%)	5.5 (7.2)	255 (31%)	5.5 (7.2)
Intellectual disability	474 (48%)	6.5 (7.0)	381 (47%)	6.5 (7.1)
OCD	79 (8%)	9.0 (7.4)	70 (9%)	9.6 (7.8)
Psychotic disorders	86 (9%)	13.9(10.2)	67 (8%)	11.7 (9.8)

*ADHD* attention-deficit/hyperactivity disorder, *OCD* obsessive–compulsive disorder

^1^HC-large if head circumference was > 38 cm. ^2^HC-small if head circumference was < 32 cm. ^3^Large for gestational age was defined as birth weight > 2 SD using the Swedish growth charts. ^4^Small for gestational age was defined as birth weight < 2 SD using Swedish growth charts. Missing values for congenital anomalies (CMA Probands/WES Probands) 335/260, large for gestational age 206/139, small for gestational age 206/139, HC-large 219/153, HC-small 219/153 individuals

### Genetic findings

Of the 808 individuals for whom WES was performed, 69 (9%) had pdSNV in an ASC102 gene, and no individuals had more than one (Additional file 4: Table S5). Of the pdSNV, 34 were predicted protein-truncating variants (frameshift, nonsense, splice acceptor or donor), and the remaining 35 were missense variants predicted to be deleterious (MPC score > 2). Genes with the highest frequency of pdSNV were *GRIN2B* (n = 6), *POGZ* (n = 5), *SATB1* (n = 4), *DYNC1H1* (n = 4), and *CREBBP* (n = 3). Two individuals had pdSNV in each of the following genes: *CACNA1E, CHD8, DIP2A, FOXP1, RORB, SETD5, STXBP1, SUV420H1* (now referred to as *KMT5B*), and *SYNGAP1*. Combining the ASC102 genes with developmental delay/ID genes from additional curated sources (ASC102 + DGR) led to the identification of 157 pdSNV in 144 probands (18%), and 12 individuals had more than one pdSNV. Using the combined list, two or more individuals had pdSNV in the following genes, not already noted above: *BRFA, CACNA1C, EHMT1, HK1, IQSEC2, KMT2A, LRP2, MTOR, PIK3CA SCN8A,* and *SMARCA4*.

Of the 996 probands who were genotyped, 105 (11%) carried one or more pdCNV (Additional file 5: Table S6). Twelve individuals had two pdCNV, for a total of 117 pdCNV overall: 66 of these were heterozygous deletions, and 51 were heterozygous duplications, ranging in size from 218 kb to 44 Mb (median 3.6 Mb). There were 59 pdCNV that were considered to be known genomic disorders (Table 3).

**Table 3:** Known genomic disorders identified based on CNV findings

Chromosomal disorder	Number identified
1p36 microdeletion syndrome	1
1q21.1 duplication syndrome	3
1q43q44 microdeletion syndrome	1
2q33.1 deletion syndrome (Glass syndrome; includes *SATB2*)	1
2q37 deletion syndrome	4
3q29 deletion syndrome	1
Cri du chat syndrome (5p15 deletion)	1
Williams-Beuren syndrome (7q11.23 deletion)	2
Jacobsen syndrome (11q deletion syndrome)	1
Prader–Willi syndrome/Angelman syndrome (15q11q13 deletion)	2
15q11q13 duplication syndrome	8
15q13.3 deletion syndrome (includes *OTUD7A* and *CHRNA7*)	3
ART-16 syndrome (16p13.3 terminal deletion)	1
16p13.11 deletion syndrome	3
16p13.11 duplication^1^	2
16p12.1 microdeletion^1^	2
16p11.2p12.2 microduplication syndrome	1
Distal 16p11.2 deletion (includes SH2B1)	1
16p11.2 deletion syndrome (proximal, BP4-BP5)	3
16p11.2 duplication syndrome (proximal, BP4-BP5)	1
Potocki-Lupski syndrome (17p11.2 duplication; includes *RAI1*)	1
17q12 duplication syndrome	2
22q11.2 deletion syndrome (Velo‐cardio‐facial syndrome/DiGeorge syndrome)	4
22q11.2 duplication syndrome	1
Distal 22q11.2 deletion (D-E)	1
Phelan-McDermid syndrome (22q13.3 deletion; includes *SHANK3*)	3
Xp22.31 deletion	2

^1^Reported in Decipher but with lesser evidence reported in ClinGen; these PDV were reclassified as not potentially damaging in the additional analysis

In the 674 probands for which there was both WES and CMA data, 123 (18%) had at least one PDV using the ASC102 gene list and the CNV list, and 182 (27%) had at least one PDV using ASC102 + DGR gene list and the CNV list.

In the PAGES data, seven individuals had a diagnosis of fragile X syndrome. Five individuals with fragile X syndrome were included in the CNV analysis (CMA probands), where one had a recurrent pdCNV (22q11.2 duplication syndrome). Three individuals with fragile X syndrome were included in the SNV analysis (WES probands), none of which had an additional pdSNV.

### Comorbidities and birth characteristics of the probands

Evaluating medical and psychiatric comorbidities among individuals with ASD (Tables 4, 5), the pdCNV and pdSNV groups showed slightly different average ages of ASD diagnosis by group and by sex, although none of these differences were significant (*p* value > 0.05). Of the phenotypes of individuals with pdCNV and pdSNV (Table 5), ID was the most common disorder.

**Table 4:** Characteristics of probands with potentially damaging CNV or SNV

	WES probands with pdSNV ASC102 list *n* (%)	CMA probands with pdCNV *n* (%)	WES and CMA probands with pdCNV or pdSNV ASC102 list *n* (%)
Total (%)	69 (9%)	105 (11%)	123 (18%)
Average diagnosis age (SD)	7.3 (7.3)	7.1 (8.1)	6.4 (7.4)
Females (n, (%))	20 (9%)	36 (12%)	35 (19%)
Average diagnosis age (SD)	8.2 (7.0)	69 (7.5)	6.2 (6.6)
Males (n, (%))	49 (8%)	69 (10%)	88 (18%)
Average diagnosis age (SD)	6.9 (7.5)	6.7 (7.5)	6.9 (7.6)

*pdCNV* potentially damaging copy number variation, *pdSNV* potentially damaging single nucleotide variation

**Table 5:** Comorbidities and birth characteristics of probands with potentially damaging CNV or SNV

Phenotypes	WES probands with pdSNV (*n* = 69) ASC102 list *n* (%)	CMA probands with pdCNV (*n* = 105) *n* (%)	WES and CMA probands with pdCNV or pdSNV (*n* = 123) AS102 list *n* (%)
HC—large^1^	4 (6%)	4 (4%)	6 (5%)
HC—small^2^	4 (6%)	13 (12%)	14 (11%)
Large for gestational age^3^	0 (0%)	2 (2%)	1 (1%)
Small for gestational age^4^	0 (0%)	7 (7%)	5 (4%)
Congenital anomalies	3 (4%)	18 (17%)	15 (12%)
Motor function disorders	2 (3%)	8 (8%)	7 (6%)
Scholastic skill disorders	11 (16%)	25 (24%)	27 (22%)
Speech/language disorders	7 (10%)	18 (17%)	16 (13%)
ADHD	11 (16%)	23 (22%)	25 (20%)
Anxiety disorder	1 (1%)	2 (2%)	2 (2%)
Epilepsy	30 (43%)	34 (32%)	49 (40%)
Intellectual disability	47 (68%)	58 (55%)	75 (61%)
OCD	3 (4%)	8 (8%)	8 (7%)
Psychotic disorders	4 (6%)	12 (11%)	14 (11%)

*ADHD* attention-deficit/hyperactivity disorder, *OCD* obsessive–compulsive disorder, *pdCNV* potentially damaging copy number variation, *pdSNV* potentially damaging single nucleotide variation

^1^HC-large if head circumference was > 38 cm. ^2^HC-small if head circumference was < 32 cm. ^3^Large for gestational age was defined as birth weight > 2 SD using the Swedish growth charts. ^4^Small for gestational age was defined as birth weight < 2 SD using Swedish growth charts. Missing values for congenital anomalies (CMA Probands/WES Probands) 335/260, large for gestational age 206/139, small for gestational age 206/139, HC-large 219/153, HC-small 219/153 individuals

For probands carrying pdSNV, versus those who did not, ID and epilepsy showed a significant positive association (Table 6), regardless of curated gene list (ASC102 vs. ASC102 + DGR). Similar patterns were observed when only considering pdSNV from the DGR list (Additional file 2: Table S7). Congenital anomalies and scholastic skill disorders were associated with carrying pdCNV (Table 7). When carriers of either pdSNV or pdCNV were assessed, ID and epilepsy showed consistent associations with PDV (Table 7).

**Table 6:** Odds ratios for comorbidities and birth characteristics of probands with potentially damaging SNV

Phenotypes	WES probands with pdSNV ASC102 list (*n* = 69)			WES probands with pdSNV ASC102 + DGR list (*n* = 144)		
	OR	95% CI	*p* value	OR	95% CI	*p* value
HC—large^1^	0.86	(0.53,5.02)	0.27	1.73	(0.67,3.97)	0.22
HC—small^2^	0.67	(0.20,1.73)	0.46	1.46	(0.76,2.64)	0.23
Large for gestational age^3^	–	–	–	0.83	(0.24,2.19)	0.73
Small for gestational age^4^	–	–	–	0.69	(0.20,1.79)	0.49
Congenital anomalies	0.57	(0.14,1.64)	0.36	0.80	(0.34,1.66)	0.58
Motor function disorders	0.51	(0.08,1.70)	0.36	0.99	(0.40,2.16)	0.99
Scholastic skill disorders	1.08	(0.52,2.05)	0.82	0.75	(0.41,1.28)	0.31
Speech/language disorders	0.77	(0.31,1.62)	0.52	0.95	(0.52,1.64)	0.85
ADHD	0.75	(0.37,1.41)	0.40	0.99	(0.61,1.57)	0.96
Anxiety disorder	0.55	(0.03,2.74)	0.56	0.56	(0.09,1.97)	0.44
Epilepsy	1.76	(1.06,2.89)	0.03*	1.51	(1.02,2.22)	0.03*
Intellectual disability	2.60	(1.55,4.48)	< 0.01*	2.62	(1.78,3.91)	< 0.01*
OCD	0.45	(0.11,1.27)	0.19	0.37	(0.13,0.85)	0.03*
Psychotic disorders	0.66	(0.20,1.66)	0.43	0.67	(0.29,1.37)	0.31

*ADHD* attention-deficit/hyperactivity disorder, *OCD* obsessive–compulsive disorder, *pdSNV* potentially damaging single nucleotide variation

^1^HC-large if head circumference was > 38 cm. ^2^HC-small if head circumference was < 32 cm. ^3^Large for gestational age was defined as birth weight > 2 SD using the Swedish growth charts. ^4^Small for gestational age was defined as birth weight < 2 SD using Swedish growth charts. Missing values for congenital anomalies 260, large for gestational age 139, small for gestational age 139, HC-large 153, HC-small 153 individuals

Significance level: *p < 0.05

**Table 7:** Odds ratios for comorbidities and birth characteristics of probands with potentially damaging CNV or SNV

Phenotypes	CMA probands with pdCNV (*n* = 105)			WES and CMA probands with pdSNV or pdCNV ASC102 list (*n* = 123)			WES and CMA probands with pdSNV or pdCNV ASC102 + DGR list (*n* = 182)		
	OR	95% CI	*p* value	OR	95% CI	*p* value	OR	95% CI	*p* value
HC—large^1^	0.97	(0.28,2.52)	0.96	1.31	(0.47,3.15)	0.58	1.42	(0.60,3.17)	0.40
HC—small^2^	1.58	(0.80,2.93)	0.16	1.45	(0.74,2.71)	0.26	1.67	(0.94,2.92)	0.07
Large for gestational age^3^	0.46	(0.07,1.54)	0.29	0.17	(0.01,0.82)	0.08	0.64	(0.21,1.61)	0.40
Small for gestational age^4^	2.05	(0.80,4.58)	0.10	1.12	(0.36,2.82)	0.83	1.82	(0.78,4.05)	0.16
Congenital anomalies	2.99	(1.61,.36)	< 0.01*	1.89	(0.96,3.56)	0.06	1.72	(0.93,3.12)	0.08
Motor function disorders	1.48	(0.63,3.07	0.33	1.27	(0.50,2.86)	0.59	1.44	(0.66,3.00)	0.34
Scholastic skill disorders	1.82	(1.10,2.92)	0.02*	1.93	(1.16,3.14)	< 0.01*	1.40	(0.87,2.21)	0.16
Speech/language disorders	1.38	(0.78,2.33)	0.25	1.08	(0.59,1.90)	0.79	1.27	(0.76,2.07)	0.34
ADHD	1.11	(0.67,1.78)	0.68	1.14	(0.69,1.83)	0.61	1.10	(0.71,1.68)	0.66
Anxiety disorder	0.59	(0.09,2.03)	0.48	0.62	(0.10,2.28)	0.54	0.62	(0.14,1.94)	0.45
Epilepsy	1.06	(0.68,0.63)	0.78	1.56	(1.04,2.33)	0.03*	1.53	(1.07,2.18)	0.02*
Intellectual disability	1.41	(0.94,2.12)	0.10	2.16	(1.14,3.24)	< 0.01*	2.37	(1.67,3.37)	< 0.01*
OCD	0.93	(0.40,1.89)	0.86	0.69	(0.30,1.43)	0.35	0.76	(0.39,1.41)	0.41
Psychotic disorders	1.40	(0.70,2.59)	0.30	1.44	(0.74,2.66)	0.26	1.10	(0.60,1.46)	0.74

*ADHD* attention-deficit/hyperactivity disorder, *OCD* obsessive–compulsive disorder, *pdCNV* potentially damaging copy number variation, *pdSNV* potentially damaging single nucleotide variation

^1^HC-large if head circumference was > 38 cm. ^2^HC-small if head circumference was < 32. ^3^Large for gestational age was defined as birth weight > 2 SD using the Swedish growth charts. ^4^Small for gestational age was defined as birth weight < 2 SD using Swedish growth charts. Missing values for congenital anomalies (CMA Probands/WES) 335/260, large for gestational age 206/139, small for gestational age 206/139, HC-large 219/153, HC-small 219/153 individuals

Significance level: *p < 0.05

We next compared the effect of pdCNV status for ASD subjects who do or do not manifest ID for the largest group of genetically characterized subjects, i.e., those who were genotyped (Table 8). We compared rates of pdCNV and phenotypes of ASD individuals with and without ID; rates were not significantly different between groups (*p* value > 0.05 for all tests). For instance, the risk for congenital abnormalities is similar for potentially damaging CNV carriers whether or not they meet criteria for ID, 2.46 versus 3.71 (Table 8), thus ID status is not driving this association..

**Table 8:** Comparison of probands with potentially damaging CNV with and without ID

Phenotypes	CMA probands without ID (*n* = 522) with pdCNV (*n* = 47, 9%)			CMA probands with ID (*n* = 474) with pdCNV (*n* = 58, 12%)		
	OR	95% CI	*p* value	OR	95% CI	*p* value
HC—large^1^	1.74	(0.39,5.48)	0.40	0.42	(0.02,2.11)	0.40
HC—small^2^	0.24	(0.01,1.19)	0.17	2.85	(1.31,5.94)	< 0.01*
Large for gestational age^3^	0.87	(0.14,3.13)	0.85	–	–	–
Small for gestational age^4^	2.05	(0.46,6.67)	0.28	2.01	(0.55,5.81)	0.23
Congenital anomalies	3.71	(1.36,9.23)	0.01*	2.46	(1.09,5.26)	0.02*
Motor function disorders	0.83	(0.13,2.94)	0.81	1.99	(0.71,4.86)	0.15
Scholastic skill disorders	2.01	(0.90,4.14)	0.07	1.61	(0.83,3.00)	0.14
Speech/language disorders	2.14	(0.92,4.54)	0.06	0.92	(0.41,1.89)	0.83
ADHD	1.43	(0.70,2.75)	0.30	0.89	(0.41,1.78)	0.76
Anxiety disorder	1.09	(0.17,4.02)	0.91	–	–	–
Epilepsy	1.21	(0.57,2.40)	0.60	0.86	(0.49,1.51)	0.61
OCD	1.50	(0.55,3.51)	0.38	0.47	(0.08,1.63)	0.31
Psychotic disorders	1.24	(0.41,3.07)	0.67	1.63	(0.63,3.68)	0.27

*ADHD* attention-deficit/hyperactivity disorder, *ID* intellectual disability, *OCD* obsessive–compulsive disorder, *pdCNV* potentially damaging copy number variation

^1^HC-large if head circumference was > 38 cm. ^2^HC-small if head circumference was < 32 cm. ^3^Large for gestational age was defined as birth weight > 2 SD using the Swedish growth charts. ^4^Small for gestational age was defined as birth weight < 2 SD using Swedish growth charts. Missing values for congenital anomalies 335, large for gestational age 207, small for gestational age 206, HC-large 219, HC-small 219 individuals

Significance level: *p < 0.05

Data for sleeping disorders, hypotonia, birth defects, prenatal growth rate, gestational age in weeks, weight and height at birth, and Apgar scores were underpowered due to a high number of missing values.

Over the course of the review, we conducted a more conservative analysis using additional criteria, in order to address questions raised during review. This resulted in removing seven individuals and reassigning 27 pdCNV as not potentially damaging, impacting 31 individuals [28 individuals in the accompanying manuscript by Klei et al. [19]] (Additional file 5: Table S6). Specifically, seven individuals were removed due to concerns about complex or recurrent PDV: One individual had very large duplications on two different chromosomes; three individuals had a terminal duplication and a terminal deletion in the same chromosome; and three individuals had an almost identical pericentromeric duplication of 13 Mb on chromosome 8. While data for all these seven individuals passed our quality control steps, we removed them in this conservative additional analysis.

Furthermore, for this additional analysis, following discussion with the Editor, the following pdCNV were reclassified as not being pdCNV: (1) large CNV in pericentromeric regions (*n* = 11); (2) for pdCNV > 1 Mb and < 3 Mb, we included only *deletions* with one or more coding exons from at least one brain-expressed gene that was *also* constrained for truncating variants (probability of loss-of-function intolerant (pLI) ≥ 0.9 in gnomAD), reclassifying 12 pdCNV as not potentially damaging; (3) CNV reported in Decipher, but with lesser evidence reported in ClinGen, specifically, two 16p13.11 duplications and two 16p12.1 were reclassified as not potentially damaging (see Table 3); (4) one large duplication in the 15q13.3 microdeletion syndrome region which met our criteria for large CNV was reclassified as not potentially damaging since duplications in this region are not considered risk loci according to DECIPHER and ClinGen. In this more conservative, supplemental analysis, two individuals had two pdCNV, for a total of 76 pdCNV across the cohort (Additional file 5: Table 6). In addition to congenital anomalies and scholastic skill disorders previously shown to be associated with individuals carrying pdCNV (Table 6), we observed associations for ID, HC-small, and small for gestational (Additional file 2: Table S8). When carriers of pdSNV and/or pdCNV were assessed, ID and epilepsy were associated with carrying PDV (Additional file 2: Table S8), similar to the previous results (Table 7).

## Discussion

Frequently, large-scale gene discovery studies are carried out on convenience samples, often with limited clinical data. Hence, the prevalence of PDV in the population cannot be readily estimated. Furthermore, while the spectrum of comorbid medical, neurological and psychiatric phenotypes for CNV has been studied extensively, less is known about comorbidities associated with pdSNV. In this study, we investigated pdSNV and pdCNV in a population sample of individuals from Sweden identified with autistic disorder. In this population sample, 27% of individuals had pdSNV (ASC102 + DGR gene list) and/or pdCNV. Carriers of pdCNV made up 11% of the individuals with autistic disorder in the Swedish population, similar to that reported for European ancestry in other studies [8, 36], while 18% of the individuals were carriers of pdSNV (ASC102 + DGR gene list). Of the 674 probands for which both WES and CMA data were available, 16 individuals had two or more PDV. Twelve individuals had two pdCNV, and five individuals had a pdCNV and pdSNV (one individual had one pdSNV and two pdCNV). One might be tempted to attribute oligogenic mechanisms for ASD on the basis of these 16 carriers; however, if PDVs occur at either a Poisson rate of 0.182 (ASC102 gene list) or 0.270 (ASC102 + DGR gene list), this number of carriers of two or more PDVs is consistent with random chance. Hence, while individuals with more than one PDV exist, and have been shown in some instances to have more severe phenotypes, our epidemiological analyses do not support what has been termed an oligogenic model in autism, i.e., where there is a nonrandom occurrence of 2 or more high-risk variants in individuals [37–39].

Consistent with prior reports [40, 41], CNV in the 15q11q13 Prader–Willi syndrome/Angelman syndrome region were most common (*n* = 10), including eight duplications and two deletions. Because we don't have access to detailed phenotype information and we could not determine the parent of origin of the deletions, we don't know if they are associated with Prader–Willi syndrome (loss of paternal allele) or Angelman syndrome (loss of maternal allele). Two other common regions in the cohort were 2q37 deletion syndrome (*n* = 4) and 22q11.2 deletion syndrome (velo‐cardio‐facial syndrome/DiGeorge syndrome) (*n* = 4), both of which are known risk factors for ASD and ID. Genes most commonly impacted by pdSNV included *GRIN2B* (*n* = 6), which is reported in individuals with ID, epilepsy, and ASD [42]. *POGZ* is emerging as a major gene in ASD [13], similar to what is observed here. Other genes with several pdSNV were *SATB1* (*n* = 4), *DYNC1H1* (*n* = 4), *SCN8A* (*n* = 3), and *CREBBP* (*n* = 3).

Some genes were impacted by either pdSNV or pdCNV. For example, *SHANK3* was disrupted in one individual by pdSNV (nonsense variant) and three individuals by pdCNV (22q13.3 deletion); *SHANK3* encodes a scaffold protein of the postsynaptic density that is essential for proper functioning of the synapse and loss of one functional copy of this gene, leading to Phelan-McDermid syndrome, has been estimated to account for ~0.5% of ASD [43]. Other ASD genes impacted by pdCNV or pdSNV include *SCN2A* (missense variant; the same variant is reported in ClinVar as de novo and likely pathogenic, variation ID: 207016) and *ASLX3* (frameshift variant).

Among individuals with autistic disorder, we observed a significant association between PDV and ID, scholastic skills disorders, and epilepsy. This association was not observed in studies with smaller sample sizes, likely due, in part, to lack of information for the comorbid conditions [44]. Individuals with pdCNV had an elevated risk for congenital anomalies, a relevant risk factor for autism. Because of the near-universal health care and national health registers in Sweden, the findings of comorbid neurological and developmental conditions were not likely to be due to ascertainment bias.

We compared the effect of pdCNV status for ASD subjects who do or do not manifest ID. ID (IQ < 70) was the most common comorbidity (47% had ID) and had sufficient sample size to make such an exploration meaningful. Although ASD subjects who had pdCNV were more likely to have ID, ASD subjects with and without ID showed no significant differences in the association of pdCNV status with other potentially associated phenotypes. Thus, conditioning on ID status does not appear to explain much of the variation for other CNV-related associations.

Research suggests epilepsy and ASD have shared etiological mechanisms [45]. A large study of 5815 children with ASD found that 12.5% had epilepsy among children aged 2–17 years, and 26% among children aged 13 years and older [46]. In the PAGES cohort, 31% of individuals with autistic disorder had epilepsy. There were multiple findings of pdSNV and pdCNV in known epilepsy genes in our study.

In the PAGES cohort, thirteen individuals had a diagnosis of Down syndrome and were not included in the current analyses. The prevalence of Down syndrome is reported to be higher for those with ASD than in the general population [47] and represent an additional genetic diagnosis for ASD in PAGES.

## Limitations

The results of this study should be interpreted in the context of some limitations. First, not all variants were validated by a second method; therefore, some could be artifacts. Nonetheless, a substantial portion of the CNV were independently validated by calling CNV from the whole exome data [31], and the validation rate of SNV is similarly high, as documented by variant calls from whole-genome versus whole-exome sequencing [48]. To further limit potentially miscalled or misclassified CNV, we went so far as to run an additional analysis, removing seven individuals with presumed pdCNV and reassigning pdCNV status for 27 pdCNV. We observed significant associations of ID, HC-small, and small for gestational with carrying pdCNV, in addition to the previously observed associations. Second, judgment calls and empirically defined thresholds were used to identify PDV. It is also important to note that this study focused on autistic disorder, and future studies on individuals with less profound ASD are warranted in order to draw a more comprehensive picture of the genetic architecture of the autism spectrum. Third, head circumference at birth, and indeed, most birth-related variables are dependent and should be interpreted with caution. Fourth, phenotypes are defined from medical registers, which may lead to under-ascertainment of comorbid diagnoses, particularly of milder findings, since the Swedish National Patient Register would not include comorbid diagnoses for those who do not seek clinical services for the relevant condition or only seek help at a primary care facility.

## Conclusions

This population survey, with its characterization of developmental impact and frequency of rare PDV, provides greater insight into the genetic architecture of ASD and associated comorbidities. pdSNV were frequent, even more frequent than pdCNV. This indicates that high-throughput sequencing is an important part of the genetic characterization of ASD. Reliable methods for calling genic CNV from sequencing data have been established [49–52]; hence, there is good reason to use sequencing as a first-tier clinical approach, especially when one considers the co-occurrence of pdSNV and pdCNV in some subjects. The high rates of genetic findings in this epidemiological cohort provide a very strong rationale for developing precision medicine approaches in ASD, with treatment tailored to differences in underlying etiology and biology. 

Rare pdCNV and pdSNV had a statistically higher occurrence in ASD subjects with ID, scholastic skill disorders, congenital anomalies, and epilepsy. These findings are consistent with prior reports, and given the nature of our sample, we can exclude ascertainment bias as the cause of this association.

Importantly, because many of the same subjects have been characterized for genotypes from common variants, we can explore the genetic architecture of ASD in even greater detail, relating common and rare variant risk. Indeed, in an accompanying manuscript by Klei et al. [19], we explore the joint contributions of rare and common variation to liability for ASD, finding that they work together approximately additively.

## Supplementary Information

**Additional file 1**: **Table S1.** List of genes used for the analysis of potentially damaging SNV

**Additional file 2**: **Table S3.** ICD codes used in this study. Table S4. Comorbidities and birth characteristics of the probands that were not genotyped or sequenced. Table S7. Odds ratios for comorbidities and birth characteristics probands with potentially damaging SNV, DGR list. Table S8. Odds ratios for comorbidities and birth characteristics of probands with potentially damaging CNV or SNV as defined for the additional analysis (see text).

**Additional file 3**: **Table S2.** List of genomic disorders derived from ClinGen and DECIPHER

**Additional file 4**: **Table S5.** List of potentially damaging SNV

**Additional file 5**: **Table S6.** List of potentially damaging CNV

## Data Availability

The data that support the findings of this study are available from the corresponding author upon reasonable request.

## Abbreviations

ACMG: American College of Medical Genetics and Genomics
ADHD: Attention-deficit/hyperactivity disorder
ASD: Autism spectrum disorder
ASC102: 102 Genes identified by the Autism Sequencing Consortium (ASC)
CI: Confidence interval
CMA: Chromosomal microarray
CNV: Copy number variation
DGR: Gene2phenotype and the Radboud University Medical Center intellectual disability gene lists
DSM: Diagnostic and Statistical Manual of Mental Disorders
gnomAD: Genome aggregation database
ICD: International classification of diseases
ID: Intellectual disability
MPC: "Missense badness, Polyphen-2, Constraint" pathogenicity score
OCD: Obsessive–compulsive disorder
OR: Odds ratio
PAGES: Population-based autism genetics and environmental study
pdCVN: Potentially damaging copy number variation
pdSNV: Potentially damaging single nucleotide variation
PDV: Potentially damaging variation
pLI: Probability of loss-of-function intolerant
SNP: Single nucleotide polymorphisms
SNV: Single nucleotide variation
WES: Whole-exome sequencing
