# Supplementary material for: Prevalence and phenotypic impact of rare potentially damaging variants in autism spectrum disorder
Source: Mol Autism. 2021 Oct 6;12:65. doi: 10.1186/s13229-021-00465-3 (PMC8495954; doi:10.1186/s13229-021-00465-3)
Supplement: Supplementary file 2 — Additional file 2: Table S3. ICD codes used in this study. Table S4. Comorbidities and birth characteristics of the probands that were not genotyped or sequenced. Table S7. Odds ratios for comorbidities and birth characteristics probands with potentially damaging SNV, DGR list. Table S8. Odds ratios for comorbidities and birth characteristics of probands with potentially damaging CNV or SNV as defined for the additional analysis (see text). [file 13229_2021_465_MOESM2_ESM.docx]

**Supplementary Information**

**Prevalence and phenotypic impact of rare potentially damaging variants in autism spectrum disorder**

**Table S3.** ICD codes used in this study

| Phenotypes | ICD-10-SE | | ICD-9-SE | ICD-8-SE |
| --- | --- | --- | --- | --- |
| Intellectual disability | |  |  |  |
| Profound, IQ below 20-25 | | F73 | 318C | 314, - |
| Severe, IQ 20-25 to 35-40 | | F72 | 318B | 313, - |
| Moderate, IQ 35-40 to 50-55 | | F71 | 318A | 312, - |
| Mild, IQ 50-55 to 70 | | F70 | 317X | 311, - |
| ADHD | | F90 | 314 |  |
| Psychotic disorders (Schizophrenia, schizotypal, delusional, and other non-mood psychotic disorders) | | F20-F29 | 295, 297, 298 |  |
| OCD | | F42.X | 300D |  |
| Anxiety disorders | | F40, F41 | 300A, 300C | 300.0, 300.2 |
| Speech/language disorders | | F80 | 80.0, 80.1, 80.2, 80.3, 80.8, 80.9, 307A, 307X, 315D, 315W, 784G,V401 | 306.00, 305.6, 306.3, 306.98, 308.-, 788.98, 781.59 |
| Scholastic skill disorders | | F81 | 81.0, 81.1, 81.2, 81.3, 81.8, 81.9, 315A, 315B, 315F, 315C, 315D, 315X, V400 | 306.1, 306.98, 315.0 - 796.0 |
| Motor function disorders | | F82 | 315D | 306.3 |
| Epilepsy | | G400-G409 | 345Y, 345K, 345L, 345M, 345MG, 345N, 345P, 345Q, 345W, 345X, 333C, 348D | 345,- |

ADHD: attention-deficit/hyperactivity disorder, OCD: obsessive-compulsive disorder

**Table S4.** Comorbidities and birth characteristics of the probands that were not genotyped or sequenced

| **Phenotypes** | **CMA but not WES probands**  **(n=323)** | |  | **WES but not CMA probands**  **(n=134)** | |
| --- | --- | --- | --- | --- | --- |
|  | **n (%)** | **Average diagnosis age**  **for ASD (SD)** |  | **n (%)** | **Average diagnosis age**  **for ASD (SD)** |
| HC – large^1^ | 13 (6%) | 5.8 (5.3) |  | 11 (10%) | 3.1 (3.3) |
| HC – small^2^ | 25 (11%) | 5.6 (6.3) |  | 3 (3%) | 4.0 (1.0) |
| Large for gestational age^3^ | 12 (5%) | 11.3 (9.8) |  | 4 (3%) | 1.5 (0.6) |
| Small for gestational age^4^ | 11 (5%) | 2.7 (3.3) |  | 9 (8%) | 3.2 (4.0) |
| Congenital anomalies | 22 (11%) | 3.1 (6.4) |  | 5 (6%) | 6.0 (9.1) |
| Motor function disorders | 23 (7%) | 5.3 (5.1) |  | 11 (8%) | 5.4 (4.4) |
| Scholastic skill disorders | 58 (18%) | 5.6 (6.0) |  | 24 (18%) | 7.1 (8.8) |
| Speech/language disorders | 50 (15%) | 6.0 (7.3) |  | 19 (14%) | 7.2 (6.8) |
| ADHD | 77 (24%) | 9.7 (7.9) |  | 34 (25%) | 7.8 (8.0) |
| Anxiety disorder | 13 (4%) | 7.8 (6.0) |  | 4 (3%) | 9.2 (8.4) |
| Epilepsy | 96 (30%) | 5.2 (6.7) |  | 42 (31%) | 5.1 (5.9) |
| Intellectual disability | 167 (51%) | 6.2 (6.6) |  | 74 (55%) | 6.4 (7.2) |
| OCD | 21 (6%) | 7.2 (5.1) |  | 12 (9%) | 9.2 (6.9) |
| Psychotic disorders | 27 (8%) | 17.3 (9.8) |  | 8 (6%) | 6.6 (4.9) |

^1^HC-large if head circumference was >38 cm. ^2^HC-small if head circumference was < 32 cm. ^3^Large for gestational age was defined as birth weight > 2 SD using the Swedish growth charts. ^4^Small for gestational age was defined as birth weight < 2 SD using Swedish growth charts

Missing values for congenital anomalies (CMA probands/WES probands) 335/260, large for gestational age 206/139, small for gestational age 206/139, HC – small 219/153, HC – large 219/153 individuals

CMA but not WES probands were the probands that were genotyped but not sequenced. WES but not CMA probands were the probands that were sequenced but not genotyped

ADHD: attention-deficit/hyperactivity disorder, OCD: obsessive-compulsive disorder

**Table S7.** Odds ratios for comorbidities and birth characteristics of probands with potentially damaging SNV, DGR list

| **Phenotypes** | **WES probands with pdSNV**  **DGR list**  **(n=127)** | | |
| --- | --- | --- | --- |
|  | **OR** | **95% CI** | **P value** |
| HC – large^1^ | 1.50 | (0.54,3.57) | 0.39 |
| HC – small^2^ | 1.72 | (0.91,3.08) | 0.08 |
| Large for gestational age^3^ | 1.15 | (0.38,2.85) | 0.78 |
| Small for gestational age^4^ | 0.95 | (0.32,2.30) | 0.91 |
| Congenital anomalies | 0.70 | (0.28,1.51) | 0.40 |
| Motor function disorders | 1.04 | (0.42,2.26) | 0.92 |
| Scholastic skill disorders | 0.86 | (0.48,1.45) | 0.58 |
| Speech/language disorders | 1.00 | (0.55,1.73) | 0.99 |
| ADHD | 0.93 | (0.57,1.49) | 0.78 |
| Anxiety disorder | 0.27 | (0.02,1.33) | 0.21 |
| Epilepsy | 1.57 | (1.06,2.31) | 0.02* |
| Intellectual disability | 3.00 | (2.01,4.55) | <0.01* |
| OCD | 0.48 | (0.18,1.04) | 0.09 |
| Schizophrenia | 0.50 | (0.19,1.10) | 0.12 |

^1^HC-large if head circumference was > 38 cm. ^2^HC-small if head circumference was < 32 cm. ^3^Large for gestational age was defined as birth weight > 2 SD using the Swedish growth charts. ^4^Small for gestational age was defined as birth weight < 2 SD using Swedish growth charts. Missing values for congenital anomalies 260, large for gestational age 139, small for gestational age 139, HC – large 153, HC – small 153 individuals

ADHD: attention-deficit/hyperactivity disorder, OCD: obsessive-compulsive disorder. Significance level: *p < 0.05

**Table S8.** Odds ratios for comorbidities and birth characteristics of probands with potentially damaging CNV or SNV as defined for the additional analysis (see text)

| **Phenotypes** | **CMA probands**  **with pdCNV**  **(n=74)** | | |  | **WES and CMA probands**  **with pdSNV or pdCNV**  **ASC102 list**  **(n=102)** | | |  | **WES and CMA probands**  **with pdSNV or pdCNV**  **ASC102 + DGR list**  **(n=162)** | | |
| --- | --- | --- | --- | --- | --- | --- | --- | --- | --- | --- | --- |
|  | OR | 95% CI | P value |  | OR | 95% CI | P value |  | OR | 95% CI | P value |
| HC – large^1^ | 1.03 | (0.24,3.00) | 0.95 |  | 1.28 | (0.42,3.25) | 0.62 |  | 1.39 | (0.56,3.15) | 0.45 |
| HC – small^2^ | 2.18 | (1.06,4.19) | 0.03* |  | 1.63 | (0.81,3.12) | 0.15 |  | 1.78 | (0.98,3.13) | 0.06 |
| Large for gestational age^3^ | 0.33 | (0.02,1.55) | 0.27 |  | 0.22 | (0.01,1.04) | 0.14 |  | 0.76 | (0.25,1.91) | 0.59 |
| Small for gestational age^4^ | 3.14 | (1.22,7.14) | 0.01* |  | 1.42 | (0.46,3.63) | 0.51 |  | 2.14 | (0.91,4.77) | 0.07 |
| Congenital anomalies | 2.93 | (1.44,5.65) | <0.01* |  | 1.56 | (0.73,3.13) | 0.24 |  | 1.45 | (0.75,2.70) | 0.25 |
| Motor function disorders | 1.87 | (0.75,4.06) | 0.14 |  | 1.59 | (0.62,3.61) | 0.29 |  | 1.68 | (0.77,3.50) | 0.17 |
| Scholastic skill disorders | 2.27 | (1.30,3.83) | <0.01* |  | 2.11 | (1.23,3.51) | <0.01* |  | 1.43 | (0.88,2.28) | 0.14 |
| Speech/language disorders | 1.65 | (0.88,2.93) | 0.10 |  | 1.15 | (0.60,2.08) | 0.65 |  | 1.32 | (0.78,2.18) | 0.28 |
| ADHD | 0.98 | (0.52,1.71) | 0.93 |  | 1.07 | (0.62,1.80) | 0.80 |  | 1.04 | (0.65,1.61) | 0.87 |
| Anxiety disorder | - | - | - |  | 0.37 | (0.02,1.90) | 0.34 |  | 0.46 | (0.07,1.70) | 0.31 |
| Epilepsy | 1.50 | (0.92,2.43) | 0.10 |  | 1.97 | (1.28,3.03) | <0.01* |  | 1.75 | (1.21,2.53) | <0.01* |
| Intellectual disability | 1.96 | (1.21,3.22) | <0.01* |  | 2.73 | (1.76,4.29) | <0.01* |  | 2.71 | (1.89,3.94) | <0.01* |
| OCD | 0.99 | (0.37,2.19) | 0.93 |  | 0.73 | (0.30,1.57) | 0.46 |  | 0.79 | (0.39,1.49) | 0.50 |
| Psychotic disorders | 1.66 | (0.77,3.23) | 0.16 |  | 1.64 | (0.82,3.09) | 0.14 |  | 1.18 | (0.63,2.11) | 0.60 |

^1^HC-large if head circumference was > 38 cm. ^2^HC-small if head circumference was < 32. ^3^Large for gestational age was defined as birth weight > 2 SD using the Swedish growth charts. ^4^Small for gestational age was defined as birth weight < 2 SD using Swedish growth charts. Missing values for congenital anomalies (CMA Probands/WES) 335/260, large for gestational age 206/139, small for gestational age 206/139, HC-large 220/153, HC-small 219/153 individuals

ADHD: attention-deficit/hyperactivity disorder, OCD: obsessive-compulsive disorder, pdCNV: potentially damaging copy number variation, pdSNV: potentially damaging single nucleotide variation

Significance level: *p < 0.05
